# Local Uses of Chloral

**Published:** 1877-04

**Authors:** 


### EXCERPTA.

Local Uses of Chloral.—In the London correspondence of the *Medical Times*, December 23d, some interesting statements are made of local applications of chloral by Dr. Dowse, Superintendent of the Highgate Infirmary. He has found it useful as an application to recent flaps after amputation. By such application there is not only relief from local pain afforded, but the unpleasant sensations felt in the extremities of the lost limbs, as in the toes, for instance, after amputation of leg, have also been avoided. When injected betwixt the flaps it relieves the, reflex startings so commonly present. The use of chloral, as an external application to sores, etc., took its origin, with Dr. Dowse, at least, in a sort of despair as to what to do in case of fungus hæmatodes of the mamma. All sorts of disinfectants had been applied without satisfactory results, so chloral was tried, with such good effects that further trial of it was made. A few cases briefly stated will illustrate its action. When applied to a large cancerous sore on the top of the head, it not only relieved the pain, but the discharge, previously most offensive, was greatly improved and rendered less offensive. In a case of cancerous ulceration of the os uteri, in combination with chloride of zinc, great relief was experienced. An impetiginoid eczema of the face, which was obstinate and painful, yielded to a solution of chloral and glycerine. The relief from pain was complete, and, in a few days, the surface began to clean, and healed rapidly. In a case of osteo-arthritis, where the pain was very intense in the knees, which were so tender that the least touch was intolerable, flannels wrung out of a hot solution of chloral gave the greatest relief.—*Western Lancet.*

